# Animal Models of Human Pathology 2020

**DOI:** 10.1155/2022/9839314

**Published:** 2022-01-22

**Authors:** Monica Fedele, Oreste Gualillo, Andrea Vecchione

**Affiliations:** ^1^Istituto di Endocrinologia ed Oncologia Sperimentale (IEOS)–CNR, 80131 Naples, Italy; ^2^Santiago University Clinical Hospital IDIS (Instituto de Investigación Sanitaria de Santiago), 15706 Santiago de Compostela, Spain; ^3^University of Rome “Sapienza”, Department of Clinical and Molecular Medicine, 00100 Rome, Italy

Studying *in vivo* the effects of new therapeutic approaches and compounds is necessary after *in vitro* research and before clinical trials. To this aim, animal models play an essential role in providing preclinical validation for efficacy and toxicity testing.

In this frame, during 2020, animal models have been vital for the current global health priority of coronavirus disease 2019 caused by severe acute respiratory syndrome coronavirus 2 (SARS-CoV-2). Indeed, neutralizing monoclonal antibodies derived from convalescence donors have been tested in Syrian hamsters to demonstrate their *in vivo* protective efficacy, opening the avenue to their use as one of the significant medical countermeasures against SARS-CoV-2 [1]. Furthermore, transgenic mice that express human angiotensin-converting enzyme 2 (ACE2) have been generated and successfully employed to test the effects of SARS-CoV-2 infection and study its pathogenicity [2].

Although animal models may show certain discrepancies with human pathophysiology, due to their great potential to resemble the complexity of the human body, well-established animal models can also represent invaluable models for the discovery of novel pathophysiological mechanisms of human disease. To cite just one example, recent evidence, performed in an experimental arthritis model used for more than 50 years, has shown that arthritis promotes a central catabolic state that can be targeted and reversed by activation of hypothalamic AMPK [3]. This might open new therapeutic alternatives to treat rheumatoid arthritis- (RA-) associated metabolic comorbidities, improving RA-patients overall prognosis.

Animal models have also represented an irreplaceable ally in outstanding research studies of the last years in the fight against cancer. Xu et al., using orthotopic tumor models in mice, demonstrated the efficacy of new personalized nano vaccines based on fluoropolymers for postsurgical cancer immunotherapy [4]. More recently, subcutaneous triple-negative breast cancer xenograft mouse models have been employed to demonstrate *in vivo* tumor targeting and antitumor efficacy of aptamer-conjugated nanovectors, capable of delivering cisplatin specifically to tumor cells [5].

In this special issue, we collected some other interesting investigations on human pathologies that have been modeled in animals to get insight into their pathogenesis and therapy. Among them, using ischemia-reperfusion rats, Żendzian-Piotrowska et al. [6] demonstrated that Tetrandrine might reduce myocardial injury by targeting the miR-202-5p/TRPV2 axis. Zhao et al. [7] used well-established rat models of cerulein-induced acute pancreatitis and streptozotocin-induced diabetes to study and compare their effect on sphingolipid metabolism in the salivary glands. The traumatic brain injury (TBI) mouse model induced by the weight-drop method was used in the study by Alqahtani et al. [8] to demonstrate the coadministration of ketamine and perampanel can reduce inflammation and, consequently, improve the behavioral function of patients with TBI. Still, in mice, Nori-Garavand et al. [9] demonstrated the positive effects of selenium on suppression of apoptosis during the vitrification-thawing process of the ovary, which is used for the preservation of fertility in humans. A piglet model was instead used by Cheng et al. [10] to demonstrate that the treatment with oral administration of resveratrol plays a beneficial role in hepatic oxidative stress and lipid balance of neonatal fatty liver diseases associated with intrauterine growth retardation.

Further studies are required to develop clinically relevant acute and chronic animal models which reflect the clinical reality of the various factors and situations that influence the biological processes of humans (age, disease, comorbidities, and gender). The closer the model is to humans, the faster it opens the door to personalized therapies.
